# Anxiety and 10-Year Risk of Incident Dementia—An Association Shaped by Depressive Symptoms: Results of the Prospective Three-City Study

**DOI:** 10.3389/fnins.2018.00248

**Published:** 2018-04-17

**Authors:** Marion Mortamais, Meriem Abdennour, Valérie Bergua, Christophe Tzourio, Claudine Berr, Audrey Gabelle, Tasnime N. Akbaraly

**Affiliations:** ^1^Institut National de la Santé et de la Recherche Médicale, Université de Montpellier, Neuropsychiatry: Epidemiological and Clinical Research, Montpellier, France; ^2^University of Bordeaux, Institut National de la Santé et de la Recherche Médicale, Bordeaux Population Health Research Center, UMR 1219, CHU Bordeaux, Bordeaux, France; ^3^Memory Resources and Research Center, Department of Neurology, Gui de Chauliac Hospital, Montpellier, France; ^4^MMDN, Université de Montpellier, EPHE, Institut National de la Santé et de la Recherche Médicale, U1198, Montpellier, France; ^5^Department of Epidemiology and Public Health, University College London, London, United Kingdom; ^6^Autism Resources Centre of Languedoc-Roussillon, University Hospital of Montpellier, CHRU de Montpellier, Montpellier, France

**Keywords:** anxiety, Spielberger State-Trait Anxiety Inventory, depressive symptoms, aging, dementia, risk factors, prospective cohort

## Abstract

**Background:** Anxiety is common in patients with cognitive impairment and dementia. However, whether anxiety is a risk factor for dementia is still not known. We aimed to examine the association between trait anxiety at baseline and the 10-year risk of incident dementia to determine to which extent depressive symptoms influence this relationship in the general population.

**Methods:** Data came from 5,234 community-dwelling participants from the Three-City prospective cohort study, aged 65 years at baseline and followed over 10 years. At baseline, anxiety trait was assessed using the Spielberger State-Trait Anxiety Inventory (STAI), and depressive symptoms using Center for Epidemiologic Studies-Depression Scale (CESD). Use of anxiolytic drugs was also considered. Diagnoses of dementia were made at baseline and every 2 years. To examine the relationship between anxiety exposures and risk of incident dementia, Cox proportional hazard regression models were performed.

**Results:** Taking anxiolytic drugs or having high trait anxiety (STAI score ≥ 44) increased the risk of dementia assessed over 10 years of follow-up [Hazard Ratio (HR) = 1.39, 95%CI: 1.08–1.80, *p* = 0.01 and HR = 1.26, 95%CI: 1.01–1.57, *p* = 0.04, respectively], independently of a large panel of socio-demographic variables, health behaviors, cardio-metabolic disorders, and additional age-related disorders such as cardiovascular diseases, activity limitations, and cognitive deficit. However, the associations were substantially attenuated after further adjustment for depressive symptoms.

**Conclusion:** Our findings suggest that depressive symptoms shape the association between anxiety trait and dementia. Further research is needed to replicate our findings and extrapolate our results to anxiety disorders.

## Introduction

Anxiety and depression are common in patients with cognitive impairment and dementia (Lyketsos et al., 2002; Geda et al., 2008; Steinberg et al., 2008; Ausén et al., 2009; Rosenberg et al., 2011). The high prevalence of those neuropsychiatric symptoms reported in cognitive impaired populations raises the question of the exact nature of their relationships and the causality link regarding age-related cognitive disorders (Livingston et al., 2017).

Depression has been extensively studied in relation to dementia and Alzheimer's disease. Even if its role-prodrome (Heser et al., 2013; Singh-Manoux et al., 2017) or risk factor (Jorm, 2000)-is still under debate, its independent association with dementia has been evidenced. In contrast, very few studies examined whether anxiety is independently associated with cognitive aging outcomes. Anxiety is often viewed as a psychological reaction to cognitive deterioration and most studies examining the anxiety-cerebral aging outcomes are cross-sectional, making an assessment of the direction of the association impossible. Amongst the few available prospective studies (Palmer et al., 2007; Devier et al., 2009; Gallacher et al., 2009; Wilson et al., 2011; Rosenberg et al., 2013), a positive association between anxiety and dementia has been reported in two studies (Wilson et al., 2011; Kassem et al., 2017), but not in Gallacher et al.'s study, carried out in general populations (Gallacher et al., 2009). Similar inconsistencies have been reported in the literature examining associations between anxiety and risk of transition to dementia in Mild Cognitive Impairment (MCI—a prodromal state of dementia) patients (Palmer et al., 2007; Teng et al., 2007; Devier et al., 2009; Ramakers et al., 2010; Rosenberg et al., 2013).

The differences in anxiety assessment, the follow-up duration generally lower than 5 years (precluding to account the preclinical phase of dementia during which dementia physiopathological processes are active), the sample heterogeneity, and the choice of adjustment variables may partly explain those inconsistent findings. In particular, depression, a frequent comorbidity of anxiety (Beekman et al., 2000; Zimmerman et al., 2000) which could share some risk factors (Roy et al., 1995; Grant et al., 2009) or even could be initiated by anxiety (Potvin et al., 2013), is not considered systematically as an adjustment factor in every study while it appears to attenuate the relationship between anxiety and risk of dementia (Gallacher et al., 2009).

The main objective of the present study was to examine the association between the risk of incident dementia and trait anxiety -a measure of individual differences in anxiety-proneness which reflects the tendency to perceive stressful situations as dangerous or threatening (Spielberger, 1983)- in an elderly general population over a 10-year follow-up period. Remaining relatively stable over time and being more related to personality than state anxiety, trait anxiety is likely to be present before the clinical manifestations of dementia and we hypothesized that it could be associated with an increased dementia risk. Additionally, because of their frequent overlap, we aimed to determine the influence of depressive symptoms in the relationship between trait anxiety and risk of dementia.

## Methods

### Participants

Participants were recruited as part of a multisite cohort study of community-dwelling older adults conducted in three French cities Bordeaux, Dijon, and Montpellier between 1999 and 2001: The Three-City (3C) Study (3C Study Group, 2003). The inclusion criteria were to be living in these cities or their suburbs and registered on the electoral rolls, to be aged 65 years and over, and not to be institutionalized. The cohort size was set at 10,000 participants (2,500 in Bordeaux, 2,500 in Montpellier, and 5,000 in Dijon) and administrative districts were selected in each city accordingly. Eligible inhabitants of the selected districts were invited to participate through a personal letter.

**Figure 1:**
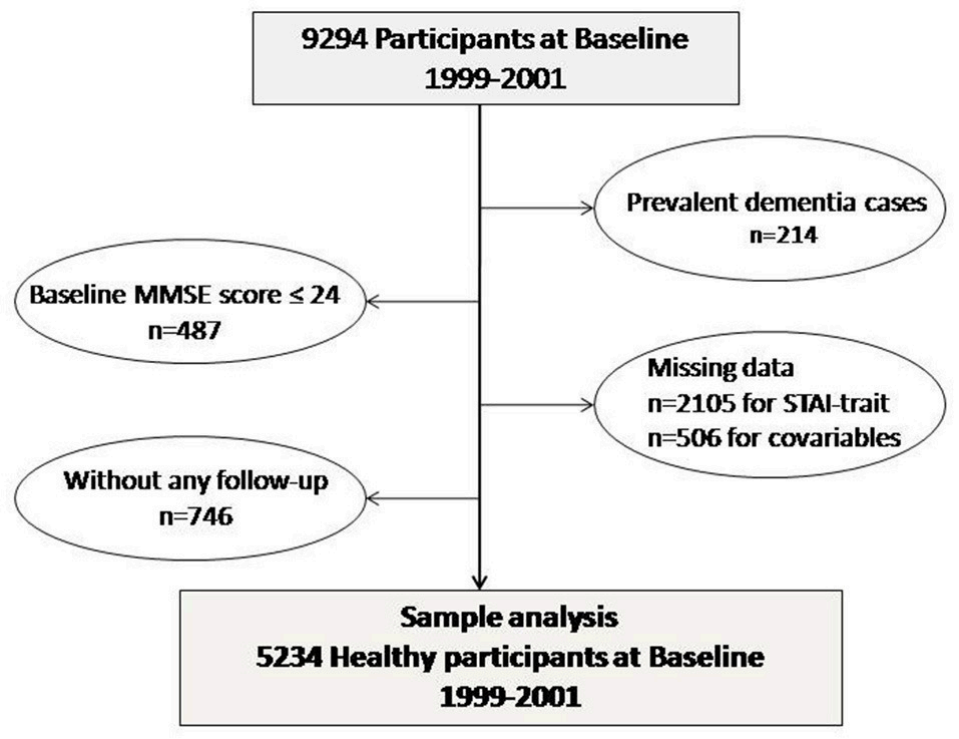
Sample analysis.

Of the 9,294 participants originally included (acceptance rate of 37%), we excluded 214 with dementia diagnosed at baseline clinical examination (1999–2001). We further excluded 487 participants with cognitive impairment assessed by a Mini Mental State Examination (MMSE) score ≤ 24 at baseline (Figure 1).

Seven hundred and forty-six participants did not have the next follow-ups (at 2, 4, 8, and 10 years after baseline). The present analyses carried out on 5,234 participants for whom complete and validated data on anxiety, depressive symptoms, dementia diagnosis, and covariates were available as detailed in the flow-chart diagram (Figure 1). Compared to participants included, the 3,357 excluded participants were significantly older and were more likely to have high score in anxiety and depressive symptoms scales and lower cognitive performances at baseline (data available on request).

#### Standard protocol approvals, registrations, and patient consents

The study protocol was approved by the Ethics Committee of the Hospital of Kremlin-Bicêtre and Sud-Méditerranée III. A written informed consent was obtained from all patients participating in the study (consent for research).

### Incidence of dementia over 10 years

At baseline, diagnosis of dementia was based on a 3-step procedure (3C Study Group, 2003; Akbaraly et al., 2009). First, trained psychologists administered a battery of neuropsychological tests detailed elsewhere (3C Study Group, 2003). Second, all the participants in Montpellier were then examined by a neurologist, whereas in Dijon and Bordeaux, because of the larger number of participants, only those who screened positive underwent further examination. Finally, an independent committee of neurologists reviewed all potential prevalent and incident cases of dementia to obtain a consensus on its diagnosis and etiology according to the criteria of the Diagnostic and Statistical Manual of Mental Disorders, fourth edition (American Psychiatry Association, 2000). Cases of Alzheimer's Disease (AD) were classified according to the National Institute of Neurological and Communicative Disorders and Stroke–Alzheimer's Disease and Related Disorders Association (McKhann et al., 1984) and cases of mixed/vascular dementia according to the National Institute of Neurological Disorders and Stroke–Association Internationale pour la Recherche en l'Enseignement en Neurosciences (Román et al., 1993).

Same procedures were performed at the next four follow-up phases (at 2, 4, 8, and 10 years after baseline) allowing to consider incident dementia screening over a total of 10 years of follow-up.

### Anxiety exposures assessed in 1999/2001

Anxiety was assessed using the Spielberger State-Trait Anxiety Inventory (STAI) (Spielberger, 1983)—a psychological inventory based on a self-report basis. The STAI consists of 40 items based on a 4-point Likert Scale and is commonly used to measure two types of anxiety -the state anxiety (S-anxiety) and the trait anxiety (T-anxiety). The T-anxiety subscale remains relatively stable over time corresponding to anxiety level as a personal characteristic and was considered alone in the present study. The total score of the T-anxiety subscale ranges from 20 to 80, with higher scores positively correlated with higher levels of anxiety symptoms.

An inventory of all drugs (prescription and over-the-counter drugs) used during the preceding month of the clinical examination was included in a standardized interview. Medical prescriptions and, where feasible, the medications themselves were checked by the interviewer. Use of anxiolytic drugs including benzodiazepine, diphenylmethane, dibenzo-bicyclo-octadiene, azaspirodecanedione derivatives, and carbamates, mephenoxalone, gedocarnil, and etifoxin (ATC codes: N05B) have then been considered.

### Covariates assessed in 1999/2001

#### Socio-demographic

Socio-demographic variables consisted of sex, age, study center, marital status (living alone/not living alone), educational level (≤ 9 years/>9 years).

#### Health behaviors

Health behaviors variables were smoking habits (never/ex or current smoker) and alcohol consumption (null/moderate if < 15 glasses of alcohol per week for women and 22 for men, or important if ≥ 15 for women and 22 for men, according to the recommendations about low and high risk alcohol consumption of the French Health High Authority (Haute Autorité de Santé, 2014).

#### Health status

Health status related covariates included vascular risk factors: body mass index (BMI) calculated from height and weight measurements performed during the clinical examination at baseline, dyslipidemia (plasma cholesterol ≥6.20 mmol/l or using lipid-lowering drugs), diabetes (glycemia ≥ 5.55 mmol/l or taking anti-diabetic treatment), hypertension (systolic/diastolic blood pressure ≥140/≥90 mm Hg or taking antihypertensive drugs) and history of vascular pathology was composed of self-reported variables and included history of stroke, angina pectoris, myocardial infarction, coronary surgery, coronary angioplasty, and arterial surgery of the legs for arteritis. Limitations in activities of daily living (IADL) using the Lawton–Brody scale (Lawton and Brody, 1969) (score > 0), and “low” cognitive performances defined by a score ≤ 27 on the MMSE have also been considered. Depressive symptoms were assessed using the Center for Epidemiologic Studies-Depression Scale (CESD) (Radloff, 1977). Participants with CESD score >16 or taking antidepressant treatment (ATC code: N06A) were considered as having depressive symptoms.

### Statistical analyses

According to the norms established in the elderly population (Bergua et al., 2012), T-anxiety subscale score was considered as high trait anxiety for scores higher than the first quartile (score ≥ 44 defined as “high trait anxiety” being compared with the score < 44, “low trait anxiety”).

Wilcoxon tests for quantitative variables and Chi2 tests for qualitative variables were used to compare characteristics of participants according to the dementia status at the end of the follow-up period.

To examine the relationship between anxiety exposures and risk of incident dementia over the 10-year follow-up period, Cox proportional hazard regression models with delayed entry (Lamarca et al., 1998) were performed with age as the basic timescale and birth date as the time origin. Results of proportional-hazard regression analyses were expressed as hazard ratios (HR) with 95% confidence intervals (CI). Participants who died or were lost to follow-up without dementia were censored at their age of death or at the last cognitive examination, respectively. The date of dementia onset was set half way between the date of the last follow-up visit when the subject was classified as normal and the date of diagnosis. We first investigated the association between anxiety exposures and risk of dementia in a model adjusted for age, sex, and center. Then we progressively entered in 2 different multivariate models the others possible confounders (education achievement, living alone, smoking habits, alcohol intake, BMI, vascular risk factors, limitations in IADL, and cognitive performances). Finally, to assess whether anxiety and depressive symptoms were independently associated with the risk of dementia, a fourth model was performed in which depressive symptoms and anxiety were simultaneously included. In addition, contribution of depression to the anxiety/risk of dementia relationship, as well as the contribution of anxiety to the depressive symptoms/risk of dementia association were examined in determining the percent attenuations (MacKinnon and Dwyer, 1993). These percent attenuations were calculated using the formula [(β_ANXIETY_ − β_ANXIETY adjusted for DEP_SYMPTOMS_)/β_ANXIETY_] × 100 and [(β_DEP_SYMPTOMS_ − β_DEP_SYMPTOMS adjusted for ANXIETY_)/β_DEP_SYMPTOMS_] × 100, where the βs are the coefficients estimated from the Cox models.

The level of statistical significance was set at *p* < 0.05. Analyses were conducted using SAS software, version 9.4 (SAS Institute).

## Results

Among the 5,234 participants with anxiety assessment at baseline, a total of 378 new cases of dementia were diagnosed over the 10 years of follow-up (57 diagnosed at the 2-year follow-up, 67 at the 4-year, 153 at the 8-year, and 101 at the 10-year follow-up period) including 259 cases of probable AD (69%), 23 cases of vascular dementia (6%), 50 cases of mixed dementia (13%), and 46 cases of other dementia types.

Seven hundred and fifty-two participants died (14%) and 1173 (22%) were lost to follow-up or refused to continue the study. Participants who were lost to follow-up were significantly older, had more frequently depressive symptomatology and anxiolytic treatment, had lower MMSE scores at baseline but did not show differences in T-anxiety subscale scores (data not shown).

Table 1 describes the characteristics of the 5,234 participants included as a function of new-onset of dementia. Compared to non-demented participants, those who developed dementia over the 10 years of follow-up were significantly older, less likely to reach a high educational achievement and more likely to live alone, to have diabetes past history of vascular diseases, depressive symptoms, IADL limitations, and lower cognitive performances. They also were more likely to have higher anxiety levels measured by the T-anxiety subscale and to use anxiolytic drugs.

**Table 1:** Baseline characteristics of the 5,234 participants as a function of new onset of dementia.

	**All participants**	**New onset of dementia cases over the 10-y of follow-up**		
	***n* = 5,234**	**Non cases *n* = 4,856**	**Dementia cases *n* = 378**	***p*-value^†^**
	***n*** **(%) or mean ± SD**	***n*** **(%) or mean ± SD**	***n*** **(%) or mean ± SD**	
**SOCIOECONOMIC FACTORS**				
Sex, women	3,069 (58.5)	2,834 (58.5)	235 (62)	0.148
Age (years)	73.4 ± 5.2	73.2 ± 5.1	76.8 ± 5.6	<0.001
**Study center**				
Bordeaux	813 (15.5)	749 (15.5)	64 (17)	0.053
Dijon	2,889 (55)	2,665 (55)	224 (59)	
Montpellier	1,532 (29.5)	1,442 (29.5)	90 (24)	
High education achievement^*^	2,139 (41)	2,010 (41)	129 (34)	0.006
Living alone	1,711 (33)	1,564 (32)	147 (39)	0.008
**HEALTH BEHAVIOR**				
**Smoking habits**				
Never/ex	4,938 (94)	4,576 (94)	362 (96)	
current	296 (6)	280 (6)	16 (4)	0.260
**Alcohol intake**				
Nul/moderate	4,301 (82)	3,982 (82)	319(84)	
High^*^	933 (18)	874 (18)	59 (16)	0.500
**HEALTH STATUS**				
Body mass index(kg/m^2^)	25.6 ± 4.0	25.6 ± 4.0	25.6 ± 4.2	0.430
Dyslipidemia^*^	2,977(57)	2,761 (57)	216 (57)	0.914
Hypertension^*^	3,953 (76)	3,659 (75)	294 (78)	0.290
Diabetes^*^	1,028 (20)	932 (19)	96 (25)	0.004
History of vascular pathology^*^	424 (8)	378 (8)	46 (12)	0.003
Cognitive impairment^*^	2,045 (39)	1,824 (38)	221 (58)	<0.001
Incapacity^*^	341 (6.5)	276 (5.5)	65 (17)	<0.001
Depressive symptomatology^*^	1,152 (22)	1,025 (21)	127 (34)	<0.001
High anxiety trait ^*^	1,672 (32)	1530 (32)	142 (38)	0.015
Use of anxiolytic drugs	738 (14)	655 (13)	83 (22)	<0.001

* *High education achievement has been defined by educational level >9 years, high alcohol intake by intake ≥15 glasses of alcohol/week for women (≥22 for men), dyslipidemia by plasma cholesterol ≥6.20 mmol/L or use of lipids lowering drugs, hypertension by systolic/diastolic blood pressure ≥140 mm Hg/≥90 mm Hg or use of antihypertensive drugs, diabetes by glycemia ≥5.55 mmol/l or antidiabetic treatment, history of vascular pathology (history of stroke, angina pectoris, myocardial infarction, coronary surgery, coronary angioplasty, and arterial surgery of the legs for arteritis), cognitive impairment by MMSE score < 27, incapacity by score>0 on the Lawton-Brody Scale, depressive symptomatology by CESD score ≥ 16 or antidepressant treatment, high anxiety trait by STAI score ≥ 44*.

† *Wilcoxon test for quantitative variables, and Chi2 test for qualitative variables*.

The comparison of characteristics of participants according to tertiles of T-anxiety subscale score showed that participants with high anxiety trait were more likely to have lower education achievement, to live alone, to be non-smokers, to have low alcohol consumption and to present dyslipidemia, lower BMI, IADL limitations, depressive symptoms, and to take anxiolytic drugs than participants with low anxiety trait (Table S1).

**Figure 2:**
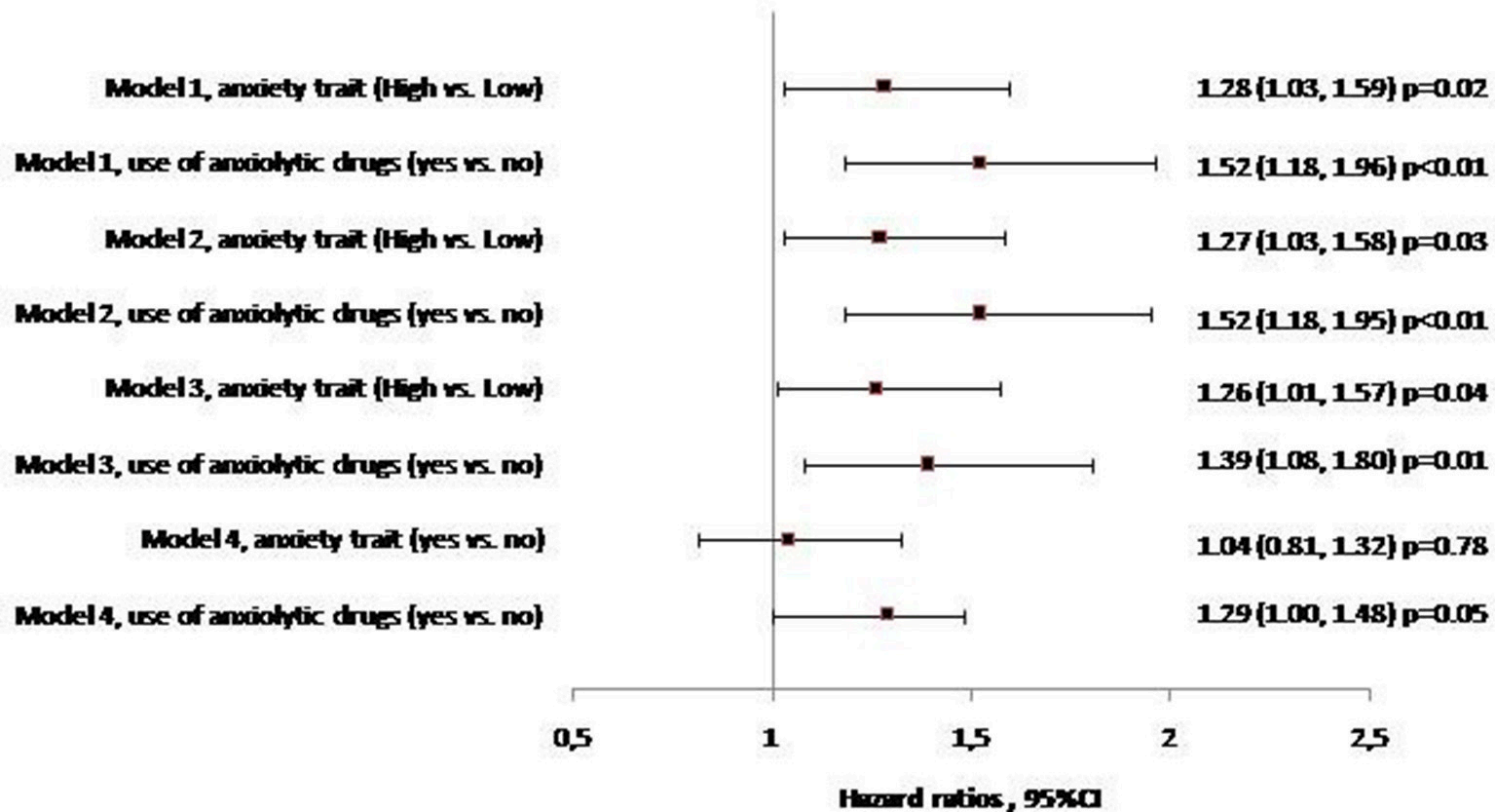
Relationship between anxiety trait, use of anxiolytic drugs and 10-year risk of incident dementia (Multivariate Cox Proportional Hazard Models, *n* = 5,234, n. event = 378). **Model 1**: Adjustment for age, sex, center. **Model 2**: Model 1 and smoking habits, alcohol intake, education, living alone. **Model 3**: Model 2 and body mass index, history of vascular pathology, hypertension, diabetes, dyslipidemia, incapacity, MMSE at baseline. **Model 4**: Model 3 and depressive symptoms.

The associations between anxiety exposure and 10-year incidence of dementia have been estimated by proportional hazards models in which both anxiety trait and use of anxiolytic drugs were included. Results are presented in Figure 2. After adjustment for age, sex, and study center, anxiety levels and use of anxiolytic drugs were both independently associated with higher risk of developing dementia. Participants with high trait anxiety showed a 28% increased risk of dementia compared to those with low trait anxiety, those under anxiolytic drugs had a 52% increased hazard ratio of dementia compared to participants not using anxiolytics. Further adjustment for education, marital status, health behaviors (smoking habits and alcohol intake), BMI, history of vascular pathology, metabolic disorder (hypertension, diabetes, dyslipidemia), incapacity and cognitive impairment (Model 2 and Model 3) had little effect on estimates (for T-anxiety scale: HR_High vs. Low_ = 1.26; 95% CI: 1.01–1.57; for use of anxiolytic drugs: HR_Yes vs. No_ = 1.39, 95% CI: 1.08–1.80).

### Joint association between anxiety exposures, depressive symptoms, and the risk of incident dementia

To further examine whether anxiety exposures and depressive symptoms were independently associated with the risk of incident dementia, a full adjusted model including simultaneously anxiety exposures and depressive symptoms was run. The T-anxiety subscale–dementia association after adjustment for depressive symptoms was substantially attenuated and did not significantly persist statistically. Similar observations were reported for the anxiolytic drugs–dementia association (Figure 2). Calculation of the percent attenuation showed that depressive symptoms attenuated the association between high trait anxiety and risk of dementia by 84.7% and the anxiolytic drugs- dementia association by 22.4% (Table 2). Conversely, high trait anxiety and use of anxiolytic drugs attenuated the association between depressive symptoms and risk of dementia by only 4.5 and 8.3%, respectively, and by 11% when included simultaneously. After adjustment for both anxiety exposures, only depressive symptoms remained significantly associated with an increased risk of dementia [β = 0.409 (0.130), *p* < 0.001].

**Table 2:** Joint association between anxiety exposures, depression and 10-year risk of incident dementia.

**Each component alone**	β adjusted (SE)^*^	***p***	
**ANXIETY EXPOSURES**			
Anxiety trait (high vs. low)	0.229 (0.112)	0.040	
Use of anxiolytic drugs (yes vs. no)	0.331 (0.131)	0.011	
**DEPRESSIVE SYMPTOMATOLOGY (YES VS. NO)**	0.555 (0.114)	<0.001	
**Joint association**	β **adjusted (SE)^†^**	***p***	**% attenuation^¶^**
Anxiety trait (high vs. low) with additional adjustment for depressive symptomatology	0.035 (0.124)	0.781	84.7%
Anxiolytic treatment (yes vs. no) with additional adjustment for depressive symptomatology	0.257 (0.132)	0.052	22.4%
Depressive symptomatology (yes vs. no) with additional adjustment for anxiety trait	0.530 (0.128)	<0.001	4.5%
Depressive symptomatology (yes vs. no) with additional adjustment for use of anxiolytic drugs	0.509 (0.117)	<0.001	8.3%
Depressive symptomatology (yes vs. no) with additional adjustment for anxiety trait and use of anxiolytic drugs	0.494 (0.130)	<0.001	11.0%

* *Adjustment for age, sex, center, smoking habits, alcohol intake, education, living alone, body mass index, history of vascular pathology, hypertension, diabetes, dyslipidemia, incapacity, MMSE at baseline*.

† *Additional adjustment for depressive symptomatology or anxiety exposures*.

¶ *The percentages by which the associations were attenuated were determined using the formula [(β_ANXIETY_ − β_ANXIETYadjustedforDEPRESSION_)/β_ANXIETY_] × 100 and [(β_DEPRESSION_ − β_DEPRESSIONadjustedforANXIETY_)/β_DEPRESSION_] × 100, where the βs are the coefficients estimated from the cox models. For instance, the percent attenuation of depression in the relationship between high STAI score and risk of incident dementia was determined using the formula [(β_STAI_ − β_STAIadjustedforDEPRESSION_)/β_STAI_] × 100, β_STAI_ = 84.7. The percent attenuation of anxiolytic treatment in the relationship between depression and risk of incident dementia was determined using the formula [(β_DEPRESSION_ − β_DEPRESSIONadjustedforANXIOLYTICTREATMENT_)/β_DEPRESSION_] × 100, β_DEPRESSION_ = 8.3*.

To further explore whether the association between anxiety exposures and dementia risk depends on the depressive symptoms, we performed supplementary analyses to assess the interaction between trait anxiety and depressive symptoms. Main analyses assessing the association between anxiety exposures and dementia risk were also repeated by stratifying them on depressive symptoms (Figure S1). Finally the risk of dementia has been compared between the following four groups of participants: no anxiety/no depressive symptoms (reference group), no anxiety/depressive symptoms, anxiety/no depressive symptoms, and anxiety/depressive symptoms, with anxiety defined as having high trait anxiety or taking anxiolytic treatment. Results reported in Figure S2 did not evidence interaction between depressive symptoms and anxiety exposures regarding dementia risk (*p* = 0.90 and *p* = 0.86, respectively).

## Discussion

The present study carried out in a large sample of elderly men and women showed that anxiolytic drugs and having high trait anxiety increased (by 40 and 25%, respectively) the risk of dementia assessed over 10 years of follow-up. Those associations were independent of a large panel of socio-demographic, health behaviors, cardio-metabolic disorders, and additional age-related disorders such as cardiovascular diseases, activity limitations, and cognitive deficit. However those anxiety exposures-dementia associations were shaped by depressive symptoms playing a strong role of confounder.

Those results are in agreement with those of the literature. In the Caerphilly dementia study (Gallacher et al., 2009), no significant association between high trait anxiety and risk of developing dementia were observed in 1,481 men followed for a 17-year period when adjusted for depression (evaluated with the 30-item general health questionnaire). However, as long as we did not consider depressive symptoms in our statistical models, our findings -showing a significant increased risk of dementia in participants with anxiety- are in line with the prospective studies in which confounding role of depressive symptoms was not considered and carried out in general populations (Wilson et al., 2011; Kassem et al., 2017) as well as in MCI patients (Palmer et al., 2007; Rosenberg et al., 2013).

In our sample, we observed that participants with a high score to T-anxiety subscale of the STAI (≥44) had a 25% higher risk of developing dementia than participants with a score < 44 independently of socio-economic, health behavior, or health status factors. However, introduction of depressive symptomatology as an adjustment factor strongly attenuated (84.7%) the relationship between trait anxiety and risk of dementia, which was no longer significant. Conversely, anxiety trait and use of anxiolytic drugs did not fundamentally change the relationship between depressive symptoms and risk of dementia, which remained significant. In addition, the absence of interaction between depressive symptoms and anxiety exposures regarding dementia risk did not support the hypothesis that the direction and the magnitude of the anxiety exposure–dementia risk association depends on the depressive symptoms. Our findings thus suggest that depressive symptomatology is a strong confounder of the anxiety-dementia relationship. In other words, when depressive symptomatology is not taken into account, the association between trait anxiety and risk of dementia may actually reflect the comorbidity of anxiety with depression. Co-occurrence of depression and anxiety are common throughout the life cycle (Regier et al., 1988; Beekman et al., 1998; Kessler et al., 2010). However, the mechanisms underlying this close relationship are still under debate. It remains unclear whether anxiety and depression share a common etiology (Roy et al., 1995; Grant et al., 2009), or whether anxiety is a prodromal stage of depression (Potvin et al., 2013). Consideration of a dimensional classification including both depression and anxiety has been suggested to be more appropriate in the elderly (Schoevers et al., 2003), and some prospective studies have even shown that the related concept of psychological distress, a mixture of anxiety and depression, was associated with AD (Wilson et al., 2003, 2005). Similarly, an overlap between anxiolytic treatment and depression is frequent. Indeed, anxiolytic drugs are commonly prescribed to people with depression in order to improve their symptoms more quickly, mitigate concomitant anxiety, and improve antidepressant treatment continuation (Bushnell et al., 2017). In our study, we observed that while the relationship between anxiety exposures and risk of dementia was substantially attenuated by depressive symptoms, anxiety exposures did not change the statistical association between depressive symptomatology and risk of dementia which remained significant. Our findings do not allow to conclude on the nature of the relationship between anxiety exposures and depression, but suggest that, whatever the nature of the link between those two entities, it is the underlying depression that drives the association between anxiety exposures and dementia. In addition, concomitant anxiety and depression do not increase the dementia risk estimated in the presence of depression alone. Taken together, those findings designate depression as the neuropsychiatric symptom to focus on in the prevention of dementia risk.

The design of the present study constitutes its main strength. Indeed, the large cohort, as well as the length of the follow-up period, provide sufficient power to estimate precisely the magnitude of the effect of anxiety trait and depression on dementia (all causes confounded) risk. The validated dementia diagnosis by an independent committee limits classification bias, while the large number of documented potential confounders, as well as the adjustment for use of anxiolytic drugs, reduce those of confounding. Limitations of our study should also be considered. Participants who were excluded from the analysis were more likely to have higher T-anxiety score at baseline and might have been at higher risk of dementia. This selection bias has probably led to an underestimation of the association reported between T-anxiety score and risk of dementia. Additionally, anxiety trait has been self-reported using the T-anxiety subscale of the Spielberger STAI. Even if its use in epidemiological framework has been validated (Spielberger, 1983), it does not capture the anxiety disorders and might reflect some aspects of depressive symptomatology. Thus, our findings on associations between anxiety trait and dementia risk in regard to depressive symptoms cannot be extrapolated to anxiety disorders.

Finally, we were not able to further conduct analyses by considering dementia subtypes (AD, vascular, and mixed dementias). Additional studies assessing the anxiety exposures, depression in relation to different dementia subtypes may provide a better understanding of anxiety, depressive symptoms and dementia relationships.

## Conclusion

In this study carried out in a large cohort of older community dwellers, we showed how associations between anxiety trait and dementia are shaped by depressive symptoms and possibly reflect the high level of comorbidity between anxiety trait and depressive symptoms. Further research is needed to replicate our findings and extrapolate our results to anxiety disorders.

## Author contributions

MM and TA: Analyzed the data and drafted the manuscript. MA: Contributed to the data analysis. AG, CB, CT, and VB: Conceived and designed the experiments.

### Conflict of interest statement

The authors declare that the research was conducted in the absence of any commercial or financial relationships that could be construed as a potential conflict of interest.
