# Supplementary material for: Anxiety and 10-Year Risk of Incident Dementia—An Association Shaped by Depressive Symptoms: Results of the Prospective Three-City Study
Source: Front Neurosci. 2018 Apr 17;12:248. doi: 10.3389/fnins.2018.00248 (PMC5913372; doi:10.3389/fnins.2018.00248)
Supplement: Supplementary file 1 [file Table1.docx]

**SUPPLEMENTARY MATERIAL**

**Anxiety and 10-year risk of incident dementia – an association shaped by depressive symptoms: Results of the prospective Three-City study**

**Contents**

[**Table 1**, **Supplemental Material**. Baseline characteristics of the study participants (n=5234) and comparison between different State Trait Anxiety Inventory (STAI) score. 1](#_Toc501313539)

[**Figure 1, Supplemental Material**. Associations between anxiety and risk of dementia according to the depressive symptomatology status of the participants over a 10-year period (n=5234, n.event=378). 3](#_Toc501313540)

[**Figure 2, Supplemental Material**. Association between anxiety/depressive symptomatology combination and risk of dementia over a 10-year period (n=5234, n.event=378). 4](#_Toc501313541)

# Table 1, Supplemental Material. Baseline characteristics of the study participants (n=5234) and comparison between different State Trait Anxiety Inventory (STAI) score.

|  | State Trait Anxiety Inventory (STAI) score. | | |  |
| --- | --- | --- | --- | --- |
|  | 1^st^ tertile  n=1785 | 2^nd^ tertile  n=1777 | 3^rd^ tertile  n=1672 |  |
| **STAI score range** | 20-34 | 35-43 | 44-77 |  |
|  | n (%) or mean ± SD | n (%) or mean ± SD | n (%) or mean ± SD | p Value† |
| **Socioeconomic factors** |  |  |  |  |
| Sex, females | 848 (47.5) | 1009 (57) | 1212 (72.5) | <0.001 |
| Age (years) | 73.6 ± 5.3 | 73.4 ± 5.2 | 73.4 ± 5.2 | 0.690 |
| Center |  |  |  |  |
| Bordeaux | 311 (17.5) | 287 (16) | 215 (13) | <0.001 |
| Dijon | 1037 (58) | 919 (52) | 933 (56) |  |
| Montpellier | 437 (24.5) | 571 (32) | 524 (31) |  |
| High education | 765 (43) | 758 (43) | 616 (37) | <0.001 |
| Living alone | 546 (30.5) | 558 (31.5) | 607 (36) | <0.001 |
| **Health behaviour** |  |  |  |  |
| Smoking habits |  |  |  |  |
| never | 974 (54.5) | 1047 (59) | 1111 (66.5) | <0.001 |
| ex | 707 (39.5) | 621 (35) | 478 (28.5) |  |
| current | 104 (6) | 109 (6) | 83 (5) |  |
| High alcohol Intake* | 361 (20) | 337 (19) | 235 (14) | **<0.001** |
| **Health status** |  |  |  |  |
| Conversion to dementia | 114 (6.5) | 122 (7) | 142 (8.5) | 0.045 |
| Body Mass Index | 26.0 ± 3.9 | 25.5 ± 3.9 | 25.3 ± 4.1 | <0.001 |
| Dyslipidemia* | 978 (55) | 1003 (56) | 996 (60) | 0.016 |
| Hypertension* | 1359 (76) | 1347 (76) | 1247 (75) | 0.539 |
| Diabetes* | 368 (21) | 357 (20) | 303 (18) | **0.154** |
| History of vascular pathology* | 145 (8) | 154 (9) | 125 (7) | 0.440 |
| MMSE at baseline ≤27 | 678 (38) | 672 (38) | 695 (42) | 0.040 |
| Incapacity* | 88 (5) | 94 (5) | 159 (10) | <0.001 |
| Depressive* symptomatology | 85 (5) | 245 (14) | 822 (49) | <0.001 |
| Use of anxiolytic drugs | 142 (8) | 203 (11) | 393 (24) | <0.001 |

*High education achievement has been defined by educational level > 9 years, high alcohol intake by intake ≥15 glasses of alcohol / week for women ( 22 for men), dyslipidemia by plasma cholesterol
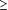
6.20 mmol/L or anticholesterol treatment plasma cholesterol
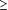
6.20 mmol/L or anticholesterol treatment, hypertension by systolic/diastolic blood pressure
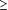
140 mm Hg /≥ 90mm Hg or antihypertensive drugs, diabetes by glycemia ≥ 5.55 mmol/l or antidiabetic treatment, history of vascular pathology by history of stroke, angina pectoris, myocardial infarction, coronary surgery, coronary angioplasty and arterial surgery of the legs for arteritis, cognitive impairment by MMSE score <27, incapacity by score>0 on the Lawton-Brody Scale, depressive symptomatology by CESD score > 16 or antidepressant treatment, anxiety trait by STAI score ≥ 44 (third tertile).

†Wilcoxon test for quantitative variables, and Chi2 test for qualitative variables

#
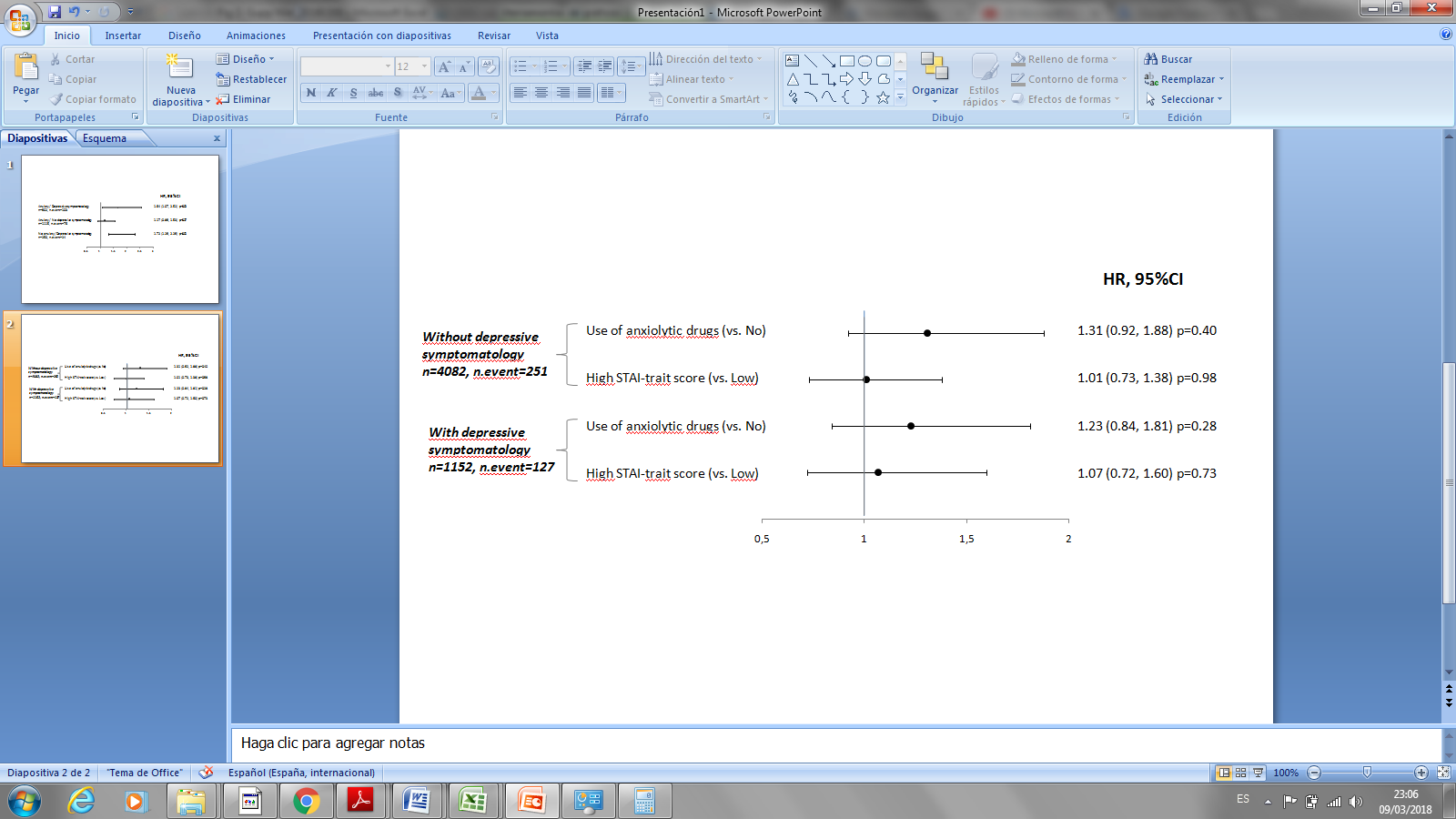


# Figure 1, Supplemental Material. Associations between anxiety and risk of dementia according to the depressive symptomatology status of the participants over a 10-year period (n=5234, n. event=378).

Hazard ratios (HR) are provided by multivariate Cox models adjusted for age sex, centre, smoking habits, alcohol intake, education achievement, living alone, body mass index, history of vascular pathology, hypertension, diabetes, dyslipidemia, incapacity, and MMSE at baseline.

#
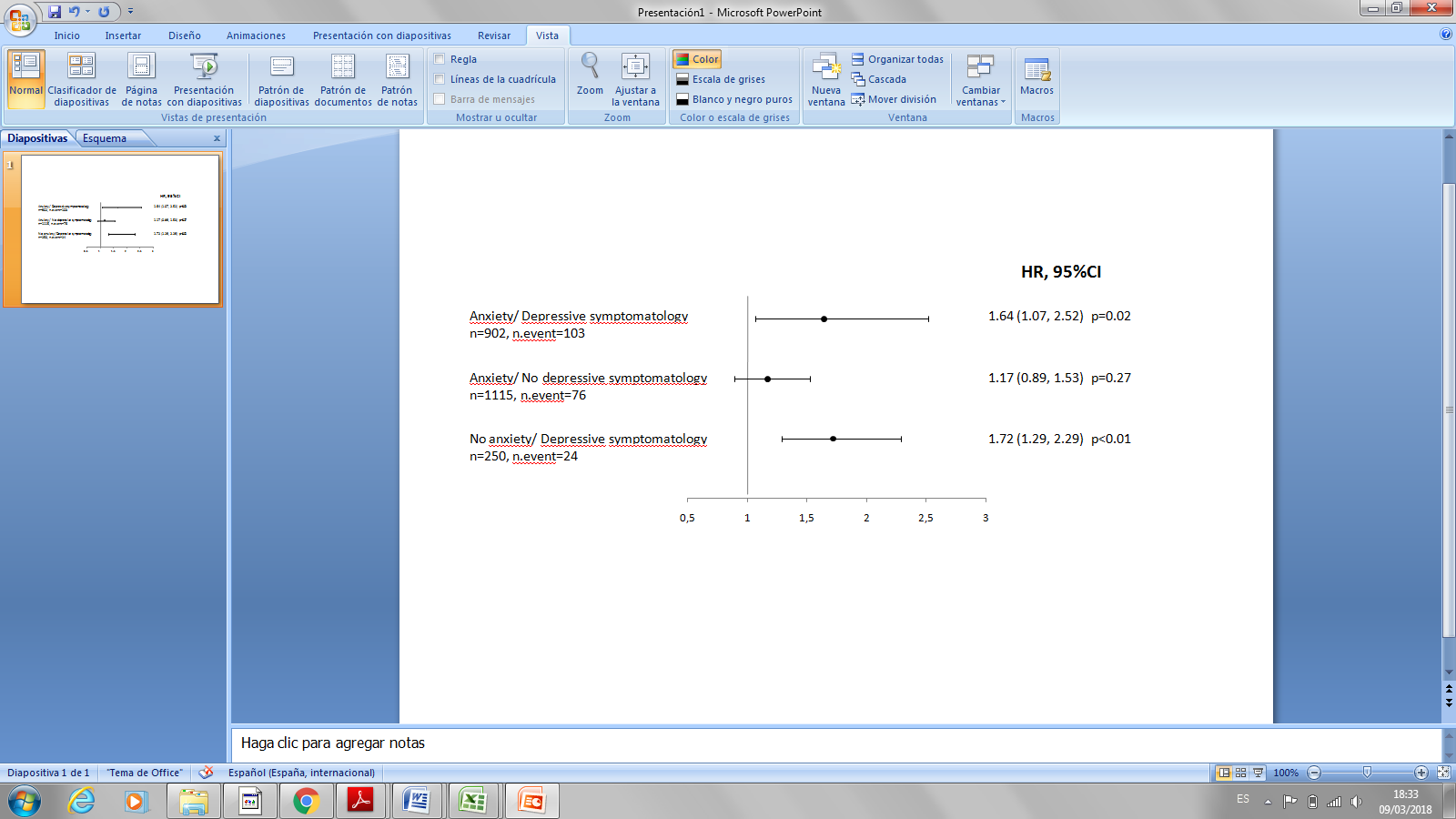


# Figure 2, Supplemental Material. Association between anxiety/depressive symptomatology combination and risk of dementia over a 10-year period (n=5234, n. event=378).

Hazard ratios (HR) are provided by multivariate Cox models adjusted for age sex, and centre. The referent category is here “no anxiety/ no depressive symptomatology”, and anxiety is defined as a high STAI-trait score (≥44) or use of anxiolytic drugs.
